# Artemisinin Reverses Glucocorticoid-Induced Injury in Bone Marrow-Derived Mesenchymal Stem Cells through Regulation of ERK1/2-CREB Signaling Pathway

**DOI:** 10.1155/2021/5574932

**Published:** 2021-09-17

**Authors:** Jiankang Fang, Marta Silva, Ruohong Lin, Wenshu Zhou, Yitan Chen, Wenhua Zheng

**Affiliations:** ^1^Centre of Reproduction, Development and Aging, Institute of Translational Medicine, Faculty of Health Sciences, University of Macau, Macau SAR, China; ^2^Jiangsu Province Key Laboratory of Anesthesiology, Xuzhou Medical University, Xuzhou, Jiangsu, China

## Abstract

Glucocorticoids are the most common cause of secondary osteoporosis, which affects both women (pre- and postmenopausal) and men. In cases of prolonged treatment, glucocorticoids promote the loss and inactivation of the differentiational function of bone marrow mesenchymal stromal cells (BMSCs), risking the development of skeletal system diseases such as osteoporosis. This study reports for the first time the protective effect of the antimalarial artemisinin against glucocorticoid-induced insults on primary cultured rat BMSCs. At relatively low concentrations, artemisinin treatment improved BMSC survival by promoting a decline of reactive oxygen species (ROS) production that correlated with the decrease of caspase-3 activation, LDH release, mitochondrial membrane potential (*Δψ*m) loss, and apoptosis induced by dexamethasone (DEXA). In addition, artemisinin improved the osteogenic differentiation of DEXA-damaged cells. DEXA inhibited extracellular-signal-regulated kinase 1/2 (ERK1/2) and cAMP response element binding protein (CREB) phosphorylation, and artemisinin treatment promoted their activation in a concentration-dependent manner. PD98059, the specific inhibitor of the ERK1/2 pathway, blocked ERK1/2 phosphorylation and artemisinin protection. Similarly, siCREB attenuated the protective effect of artemisinin, strongly suggesting the involvement of the ERK1/2-CREB pathway in the protective action of artemisinin against DEXA-induced damage in BMSCs. In addition, we found that the expression of antiapoptotic protein B-cell lymphoma 2 protein (BCL-2) was also upregulated by artemisinin. These studies demonstrate the therapeutic potential of artemisinin in the survival improvement of BMSCs exposed to glucocorticoid-induced apoptosis and suggest that artemisinin-mediated protection may occur via the activation of ERK1/2-CREB signaling pathway.

## 1. Introduction

Bone marrow mesenchymal stromal cells (BMSCs) are among the most promising seed cells used in tissue engineering approaches and whose potential for the treatment of different conditions [1], especially bone diseases, has been widely explored [2]. Osteoporosis (OP) is a common and very serious systemic bone metabolic disease that can be due to congenital factors (idiopathic OP) or induced by the decline of estrogen levels in menopausal women, by certain diseases, such as diabetes, or certain therapies, such as the long-term use of high concentrations of glucocorticoids.

Currently, glucocorticoids are the drug of choice for the treatment of many chronic and autoimmune diseases, such as rheumatoid arthritis [3], connective tissue diseases [4], inflammatory bowel disease [5], acute respiratory syndrome [6], chronic lung diseases [7], asthma [8], and also in organ transplantation [9]. According to the statistics, 1-2% of the worldwide population suffering from different diseases and in different age groups receive long-term glucocorticoids therapy [10]. However, the prolonged use of high-dose glucocorticoids has been associated with serious adverse reactions, including bone loss that results in OP and affects up to 50% of patients [11, 12]. In recent years, glucocorticoid-induced OP (GIOP) has become the most common form of secondary OP, as well as a common type of OP in young people [13]. Although the underlying mechanisms are not entirely clear, many factors are known to be involved in the pathogenesis of GIOP. At the cellular level, the mechanism of GIOP is related to an imbalance in the bone remodelling function, which involves the bone-forming osteoblasts (OB) and the bone-resorbing osteoclasts (OC) [14]. OB are derived from the differentiation of BMSCs, which encompasses three stages: cell proliferation, matrix maturation, and matrix mineralization. During the proliferation stage, extracellular matrix proteins are expressed and secreted by osteoblasts, resulting in the formation of nonmineralized bone matrix and osteoid. Then, the osteoid proteins crosslink during the matrix maturation stage, forming a stronger and more stable structure. Crosslinked collagen type I fibrils, the main component of the osteoid, subsequently become a template in which the inorganic minerals are deposited to form the mineralized bone matrix (MBM). Finally, the osteoblasts are embedded in the bone as osteocytes and become bone-lining cells or undergo apoptosis [15, 16].

BMSCs are very important for the maintenance of bone health. These cells not only supplement the number of osteoblasts but also secrete endogenous growth factors in the form of paracellular secretion, further promoting the osteogenesis process. Hence, protecting BMSCs from damage is essential for bone health, especially for OP prevention. At physiological concentrations, glucocorticoids can support the osteogenesis and chondrogenesis differentiation of BMSCs [17, 18]. However, high concentrations can cause damage to BMSCs, disrupting the balance between osteogenic and adipogenic differentiation and the balance of OB-mediated bone formation and OC-mediated bone resorption. Under these conditions, BMSC differentiation toward adipocytes may increase and toward osteoblasts may decrease, resulting in bone loss and fat accumulation. In fact, it was previously reported that GIOP patients not only lose bone but also accumulate marrow adipose tissue, which is indicative of glucocorticoid-induced alterations in the lineage commitment of BMSC differentiation into adipocytes at the expense of osteoblasts [19–21]. In another study, glucocorticoid-induced damage to BMSCs resulted in the inhibition of BMSC proliferation and in the increase of apoptosis [22]. During the process of glucocorticoid damage to BMSCs, autophagy was reported to be stimulated in the cells, protecting them from apoptosis. In fact, autophagy inhibition has been reported to increase apoptosis, further indicating that the occurrence, to a certain extent, of glucocorticoid-mediated autophagy while inducing apoptosis of BMSCs may protect BMSCs from glucocorticoid-induced apoptosis. However, this self-protective process is not enough to protect most cells from apoptosis, nor can it change the state of greatly reduced cell viability.

We previously reported that artemisinin, a first-line antimalarial drug derived from the Chinese herb *Artemisia annua L*, protected BMSCs from H_2_O_2_-induced apoptosis via activation of the ERK signaling pathway [23]. In that study, we also found that artemisinin prevented H_2_O_2_-induced BMSCs' osteogenic differentiation inhibition. This study is aimed at assessing the protective effect of artemisinin against glucocorticoid-induced oxidative stress injury in BMSCs and at exploring the potential underlying mechanisms of action in order to provide a theoretical basis for the treatment or prevention of GIOP. Up to now, many studies have confirmed that high doses of glucocorticoids can cause the intracellular oxidative stress levels to rise and cause cell damage, thereby inducing apoptosis [24–27]. However, no study has been reported that glucocorticoids cause oxidative stress in BMSCs. In this study, we used a high dose of glucocorticoids to induce BMSC apoptosis and investigated whether glucocorticoid-induced apoptosis was associated with an increase of oxidative stress levels. In addition, the protective effect of artemisinin on glucocorticoid-induced BMSC apoptosis was assessed. Interestingly, we found that high doses of glucocorticoids could, indeed, trigger an increase in ROS levels and apoptosis. Pretreatment of these BMSCs with artemisinin had a protective effect against glucocorticoid-induced reduction of cell viability and apoptosis. In addition, artemisinin prevented the decrease of mitochondrial membrane potential (*Δψ*m) and the increase of ROS levels induced by glucocorticoids. Importantly, we also observed that high concentrations of glucocorticoids inhibited the osteogenic differentiation of BMSCs, which was reversed to some extent by artemisinin. Investigation of the mechanisms underlying the protective effect of artemisinin revealed that it may occur via the ERK1/2-CREB signaling pathway. This study provides a new protective approach for BMSCs from glucocorticoid-induced apoptosis and new insights into potential future GIOP therapies.

## 2. Materials

DMEM/F12 medium was purchased from Thermo Fisher Scientific, Waltham, MA, USA; dexamethasone and propidium iodide were bought from Meilun Biotech Co. Ltd., Dalian, China; 3-(4,5-dimethylthiazol-2-yl)-2,5-diphenyltetrazolium bromide (MTT) and Hoechst 33342 solution were obtained from Molecular Probes, Eugene, OR, USA; CellROX™ Green Reagent was obtained from Thermo Fisher Scientific, Waltham, MA, USA; TUNEL kit, Caspase-3 Activity Assay Kit, and mitochondrial membrane potential assay kit with JC-1 were purchased from Beyotime Biotechnology, Haimen, China; Annexin V-FITC/PI Kit was obtained from Sangon Biotech, Shanghai, China; artemisinin and dimethyl sulfoxide (DMSO) were received from Sigma-Aldrich, St. Louis, MO, USA; BCA protein assay kit was obtained from Beyotime Institute of Biotechnology, Beyotime, Beijing, China; anti-ERK1/2, anti-phospho-ERK1/2, anti-phospho-CREB, anti-Bcl-2, anti-AKT, and anti-*β*-actin were purchased from Cell Signalling Technology (CST), Woburn, MA, USA; anti-GAPDH was bought from Beyotime, Beijing, China; Clarity Western ECL substrate was bought from Bio-Rad, Hercules, CA, USA; MEK/ERK inhibitor PD98059 was obtained from Merck Millipore, Darmstadt, Germany; siCREB was obtained from GenePharma, Shanghai, China; lipofectamine RNAiMAX was obtained from Invitrogen, Carlsbad, CA, USA; fetal bovine serum (FBS) was obtained from GIBCO, Grand Island, NY, USA; and Alizarin Red S was received from Sigma-Aldrich, St. Louis, MO, USA.

## 3. Methods

### 3.1. MTT Assay for Cell Viability Detection

BMSCs were seeded on 96-well plates at a density of 3-5 × 10^4^ cells/mL in 1% serum medium as previously described [23]. For detecting glucocorticoid cytotoxicity, BMSCs were incubated with different concentrations of dexamethasone (DEXA, 0.1–30 *μ*M) in serum-free medium for 24 h or 72 h, followed by incubation of MTT (0.5 mg/mL) for 2 h. To determine the protective effect of artemisinin from DEXA-induced cellular damage, the cells were incubated with different doses of artemisinin (0.1-3.0 *μ*M) for 1 h. Then, the medium with artemisinin was discarded, and DEXA (30 *μ*M) was added to the wells for 24 h at 37°C. After this period, MTT (0.5 mg/mL) was added and incubated for 2 h. The medium containing MTT was then discarded, and 100 *μ*L of DMSO was added into each well. The optical density (OD) values were read at a 570 nm wavelength after 10 min of shaking using an Infinite M200 PRO Multimode Microplate Reader (Tecan, Männedorf, Switzerland). The viability of living BMSCs was calculated as a percentage of the control group.

### 3.2. PI Staining for Cell Viability Analysis by FACS

PI staining for FACS analysis was used to assess BMSC viability as previously described [23]. Briefly, BMSCs were seeded on 12-well plates at a density of 1-2 × 10^5^ cells/mL. After appropriate treatment, BMSCs were harvested and washed twice with PBS. Then, the cells were resuspended in the binding buffer at a density of 2 × 10^5^ cells/mL. 2 *μ*g/mL PI was added into the resuspended cells followed by 15 min incubation on ice protected from light. The PI fluorescence was determined with a BD Accri C6 Plus. Collected data were analysed by the software of BD Accri C6 Plus.

### 3.3. TUNEL/DAPI Costaining for Apoptosis Assessment

Terminal deoxynucleotidyl transferase dUTP nick end labelling (TUNEL) staining (green fluorescence) was used to measure the cellular apoptosis, and DAPI staining (blue fluorescence) was applied to count the total number of BMSCs. TUNEL/DAPI costaining was performed using the TUNEL kit and DAPI as previously described [28] with minor modifications. Briefly, the processed samples were incubated with a TUNEL reaction mixture for 1 h at 37°C protected from light. After that, BMSCs were colabelled with DAPI solution (final concentration: 1 mg/mL) for 5 min. Finally, cells were washed and visualized by EVOS FL Imaging System (Thermo Fisher Scientific, Waltham, MA, USA). Apoptotic cells were identified as having the nucleus with green colour, and the percentage of apoptotic BMSCs was calculated from the total nucleus population. The percentage of apoptotic BMSCs was analysed by the ImageJ software.

### 3.4. Annexin V-FITC/PI Assay for Apoptosis Assessment Analysed by FACS

The apoptosis of BMSCs was analysed as previously described [23] with minor modifications using the FACS methodology after staining with Annexin V-FITC/PI. Briefly, BMSCs were plated in 6-well plates (2-3 × 10^5^ cells/mL). After appropriate treatments, adherent BMSCs were harvested and washed. Then, the cells from each well were suspended in 195 *μ*L of binding buffer at a density of 2 × 10^5^ cells/mL and incubated with Annexin V-FITC (10 *μ*g/mL, 5 *μ*L) at room temperature for 15 min protected from light. Finally, BMSCs were washed with the binding buffer and centrifuged at 1000 rpm for 5 min. BMSCs were suspended in 190 *μ*L of binding buffer with 10 *μ*L of PI (20 *μ*g/mL), and BD Accri C6 Plus was used for data acquisition. Collected data were analysed by the BD Accri C6 Plus software. The apoptosis rate of BMSCs was expressed as the percentage of Annexin V-positive cells.

### 3.5. Caspase-3 Activity Assay for Apoptosis Analysis

Caspase-3 activity was analysed using the Caspase-3 Activity Assay Kit according to the manufacturer's protocol as previously described [23]. Briefly, BMSCs were seeded in 6-well plates (3-5 × 10^4^ cells/mL) and appropriately treated. Afterwards, both the medium and cells were collected and centrifuged for 5 min at 4°C (600 g). Collected cells were resuspended and incubated in lysis buffer on ice for 15 min and then centrifuged for another 12 min at 4°C (18000 g). Then, the cell lysate supernatant was collected. 100 *μ*L of reaction mixture containing 40 *μ*L of assay buffer (supplied with the kit), 50 *μ*L of cell lysate supernatant, and 10 *μ*L of caspase-3 substrate Ac-DEVD-pNA (2 mM) (supplied with the kit) was incubated at 37°C for 2 h. Finally, caspase-3 activity was measured at the wavelength of 405 nm using the Infinite M200 PRO Multimode Microplate Reader. The percentage of caspase-3 activity was calculated compared to the control group.

### 3.6. Measurement of Mitochondrial Membrane Potential (*ΔΨ*m)

The decline of *Δψ*m is considered an important event in early cellular apoptosis [29]. Therefore, *ΔΨ*m was measured as previously published [23] using the JC-1 assay kit. Briefly, BMSCs were seeded in 6-well plates (3-5 × 10^4^ cells/mL) and appropriately treated with the drugs. The analysis of *ΔΨ*m was performed based on the guidelines provided in the JC-1 kit. Images were taken using the EVOS FL Imaging System. The ratio of red/green fluorescence intensity was calculated by the ImageJ software.

### 3.7. Measurement of Cellular Oxidation

CellROX® Oxidative Stress Reagents are fluorogenic probes used to measure the reactive oxygen species (ROS) in live cells. Accordingly, ROS were evaluated by using these probes as previously described [30] with minor modifications. Briefly, after appropriate drug treatments, BMSCs were incubated for 30 min at 37°C with CellROX® Deep Green reagent. Then, BMSCs were washed three times with PBS, and images were taken using the EVOS FL Imaging System. Semiquantification of the ROS levels was performed using ImageJ, and the oxidative stress levels (%) were calculated as a percentage of the control group.

### 3.8. Detection of p-CREB Expression Using Immunofluorescence

The expression of p-CREB in BMSCs was detected by immunofluorescence (IF). After appropriate treatment, BMSCs were fixed with 4% paraformaldehyde solution at room temperature for 30 min. After being washed with PBS three times, BMSCs were permeated by 0.5% Triton X-100/PBS solution for 30 min at room temperature. Then, the cells were incubated with a blocking buffer (3% BSA) for 60 min at room temperature, followed by incubation with primary antibody (p-CREB, 1 : 200) overnight at 4°C. In the next day, the cells were washed three times with PBS and incubated with secondary antibody for 1 h at room temperature. After washing the cells with PBS, images were taken using the EVOS FL Imaging System.

### 3.9. CREB Silencing by siCREB Transfection

Gene silencing of CREB was performed using specifically synthesized siRNA. Corresponding mRNA sequences for the siRNAs were as follows: siCREB-rat-647, 5′-GCCUGCAGACAUUAACCAUTT-3′; siCREB-rat-744, 5′-GCCCAGCAACCAAGUUGUUTT-3′; siCREB-rat-129, 5′-GCCACAGAUUGCCACAUUATT-3′; and negative control siRNA, 5′-UUCUCCGAA CGUGUCACGUTT-3′. BMSCs were grown in DMEM/F12 medium supplemented with 10% FBS, 100 units/mL penicillin, and 100 *μ*g/mL streptomycin. For CREB silencing, BMSCs were transfected with siCREB using lipofectamine RNAiMAX (Invitrogen) for 48 h, based on the manufacturer's protocol.

### 3.10. Analysis of Osteogenic Differentiation with DEXA and Artemisinin Coculture

Osteogenic differentiation of BMSCs was performed using the prepared osteogenic differentiation medium as previously described [23]. Briefly, cells were seeded in 12-well plates at a density of 3-5 × 10^4^ cells/mL. After reaching 60% confluency, the culture medium was replaced by the osteogenic differentiation medium consisting of DMEM, 10% FBS, 1% penicillin-streptomycin, 1% L-glutamine, 0.1 *μ*M DEXA, 50 *μ*M ascorbic acid, and 10 mM glycerol 2-phosphate (BGP). The induction medium was changed every 3 days for 21 days. During the osteogenic differentiation induction process, cells were cocultured with different concentrations of DEXA and artemisinin. After induction, the cells were washed twice with PBS followed by fixation with 4% paraformaldehyde (PFA) solution for 20 min at 25°C. The cells were then stained with 2% alizarin red S (ARS) staining solution for 25 min at room temperature to identify the calcium deposits.

### 3.11. Western Blotting

For the detection of protein expression levels, Western blotting (WB) was carried out as previously described [31]. Briefly, cells were rinsed with PBS and lysed by RIPA buffer at 4°C. The concentration of the total proteins was determined using the BCA protein kit. The proteins were separated by polyacrylamide gel electrophoresis and electro-transferred to PVDF membranes. Membranes were incubated with 3% (*w*/*v*) BSA in TBST (TBS with 0.1% Tween 20) for 1 h at room temperature and then incubated with the corresponding primary antibodies at 4°C overnight. In the next day, the membranes were washed with TBST and incubated at room temperature for 1 h, with the secondary antibodies conjugated with horseradish peroxidase. The membranes were then washed 3-5 times with TBST, and the bands were visualized using Clarity Western ECL substrate.

### 3.12. Statistical Analysis

All experiments were performed three times, and the data are expressed as mean ± standard deviation (SD). One-way ANOVA followed by Tukey's multiple comparison was used in the statistical analysis using Graph Pad Prism 5.0 (Graph Pad Software Inc., USA), and the value *p* < 0.05 was considered statistically significant.

## 4. Results

### 4.1. DEXA Induced Cellular Death in BMSCs

**Figure 1:**
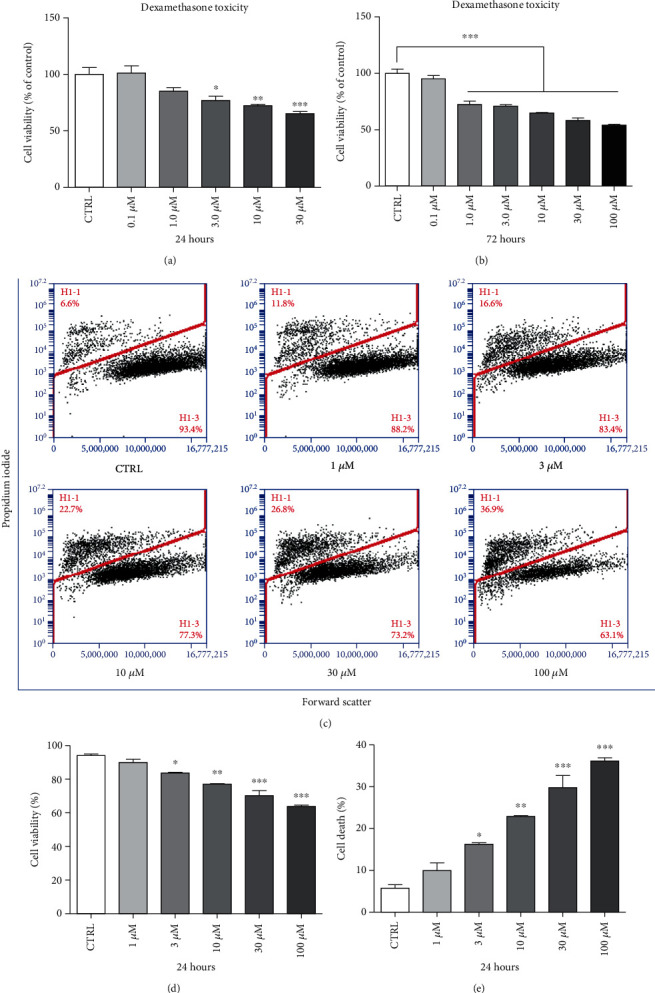
The toxicity of DEXA in BMSCs. (a, b) BMSCs were cultured with different concentrations of DEXA or DMSO (1%, vehicle control, CTRL) for 24 h or 72 h, and the cell viability was assessed using the MTT assay (*n* = 4). (c) BMSCs were cultured with different concentrations of DEXA or DMSO (1%, vehicle control, CTRL) for 24 h; the cell viability was detected by FACS using PI staining (*n* = 4). (d) Cell viability analysis of (c). (e) Cell death analysis of (c). ^∗^*p* < 0.05, ^∗∗^*p* < 0.01, and ^∗∗∗^*p* < 0.001 were considered significantly different. CTRL: control.

BMSCs were incubated with different concentrations of DEXA for different time periods to evaluate which conditions were more efficient for inducing BMSC apoptosis. Analysis of cell viability using MTT assay showed that DEXA reduced the survival rate of BMSCs. When cultured for 24 h and 72 h, DEXA significantly inhibited the proliferation of BMSCs in a dose-dependent manner starting at 3.0 *μ*M (24 h) and 1.0 *μ*M (72 h) (Figures 1(a) and 1(b)). PI staining analysis showed that DEXA caused necrosis of BMSCs in a dose-dependent manner. BMSCs cultured with 1.0 *μ*M DEXA for 24 h showed no significant differences in cell survival or mortality rates compared to the control group. However, starting from 3.0 *μ*M DEXA, there was a significant decrease in the survival rate and an increase in the mortality rate compared to the control group. Thus, the results of PI staining are consistent with the obtained MTT results (Figures 1(c)–1(e)).

### 4.2. Artemisinin Attenuated DEXA-Induced Cell Viability Loss

**Figure 2:**
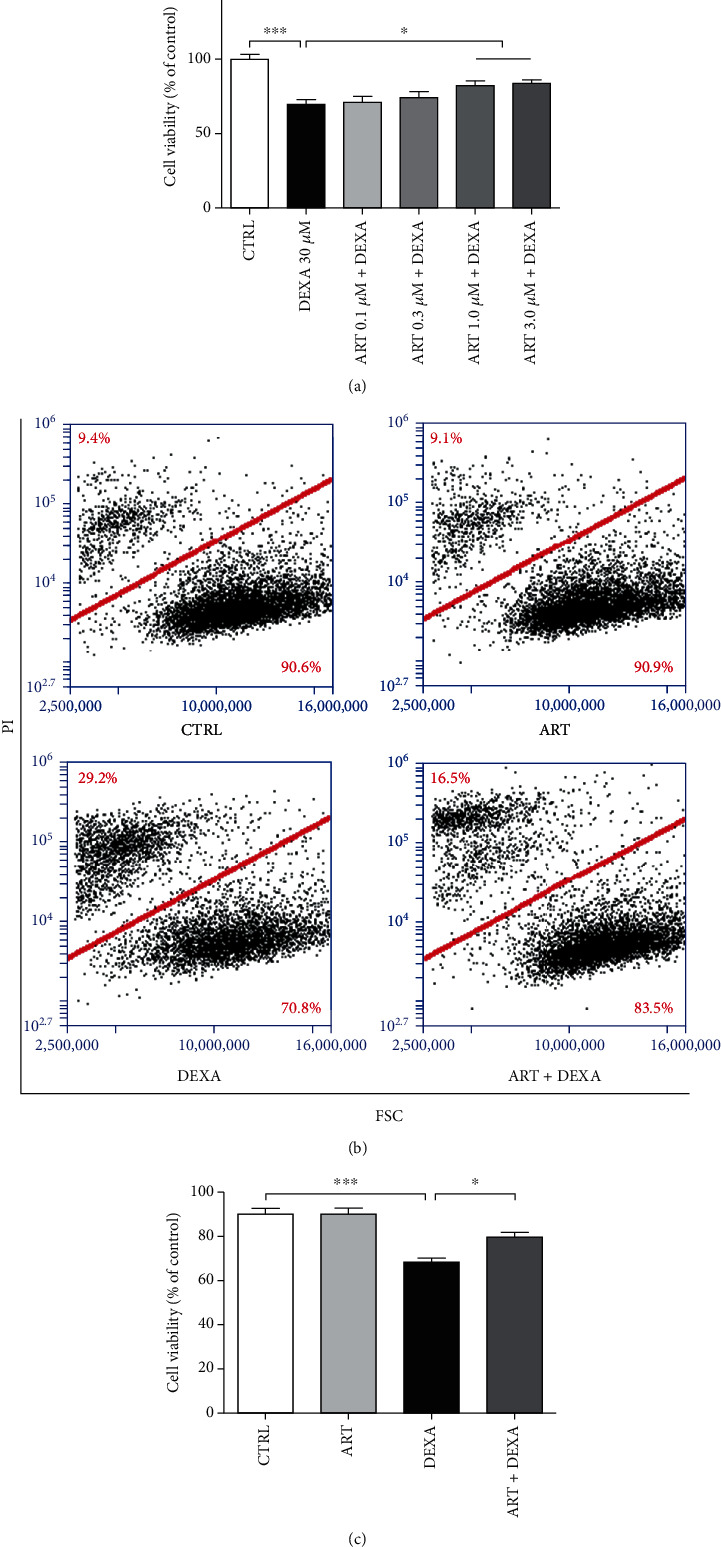
Artemisinin attenuated the decrease of cell viability induced by DEXA. (a) BMSCs were pretreated with different concentrations of artemisinin (ART) or DMSO (1%, vehicle control, CTRL) for 1 h before incubation with 30 *μ*M DEXA for another 24 h; the cell viability was detected by MTT assay (*n* = 4). (b) BMSCs were pretreated with artemisinin (ART, 1.0 *μ*M) or DMSO (1%, vehicle control, CTRL) for 1 h before incubation with 30 *μ*M DEXA for another 24 h; the cell viability was detected by FACS using PI staining (*n* = 4). (c) Quantification of (b) (*n* = 4). ^∗^*p* < 0.05 and ^∗∗∗^*p* < 0.001 were considered significantly different. CTRL: control; ART: artemisinin; DEXA: dexamethasone; ART + DEXA: treated with artemisinin followed by incubation with DEXA.

After determination of the optimal concentration of DEXA for inducing BMSC death, the cells were pretreated with different concentrations of artemisinin for 1 h and then incubated with 30 *μ*M DEXA for another 24 h. PI staining and MTT assay were used to evaluate the protective effect of artemisinin against DEXA-induced cell viability loss. Obtained MTT results showed that artemisinin prevented the decrease of DEXA-induced BMSC survival rate in a dose-dependent manner. This effect was significant in cells pretreated with 1.0 and 3.0 *μ*M artemisinin (Figure 2(a)). Consistent with these findings, PI staining FACS results showed that 1.0 *μ*M artemisinin significantly antagonized DEXA-induced cell viability loss (Figures 2(b) and 2(c)).

### 4.3. Annexin V-FITC/PI Staining Assay: Artemisinin Attenuated DEXA-Induced Apoptosis

**Figure 3:**
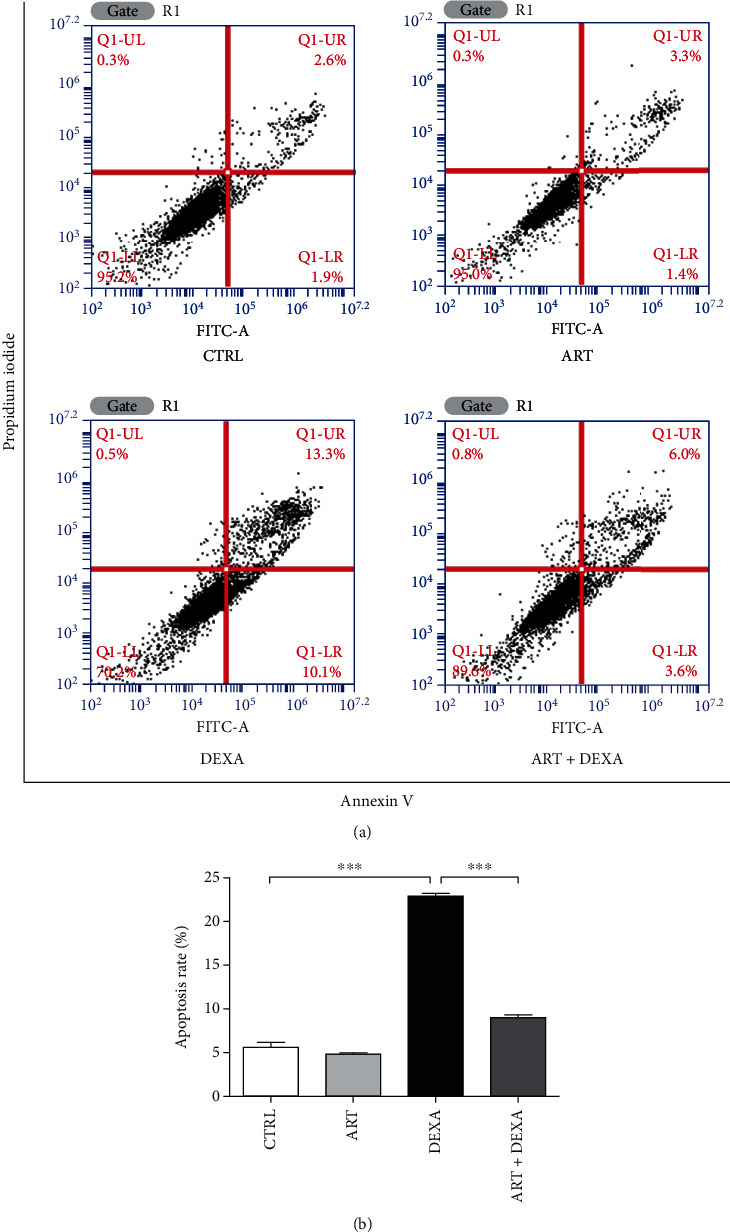
Artemisinin attenuated DEXA-induced apoptosis detected by Annexin V-FITC/PI staining in FACS. After pretreatment with artemisinin (1.0 *μ*M) or DMSO (0.1%, vehicle control) for 1 h, BMSCs were incubated with 30 *μ*M DEXA for another 24 h. (a) The cellular apoptosis was analysed by Annexin V-FITC/PI staining, detected by FACS (*n* = 4), and quantified by the apoptosis rate (b) (*n* = 4). ^∗∗∗^*p* < 0.001 was considered significantly different. CTRL: control; ART: artemisinin; DEXA: dexamethasone; ART + DEXA: treated with artemisinin followed by exposure to dexamethasone.

Assessment of the protective effect of artemisinin on DEXA-induced cellular apoptosis using Annexin V-FITC/PI staining detected by flow cytometry revealed that artemisinin significantly attenuated DEXA-induced apoptosis and significantly reduced the rate of apoptotic cells (Figure 3). These results are indicative that artemisinin pretreatment had a protective effect against DEXA-induced apoptosis of BMSCs.

### 4.4. TUNEL/DAPI Costaining and Caspase-3 Activity Assays: Artemisinin Attenuated DEXA-Induced Apoptosis

**Figure 4:**
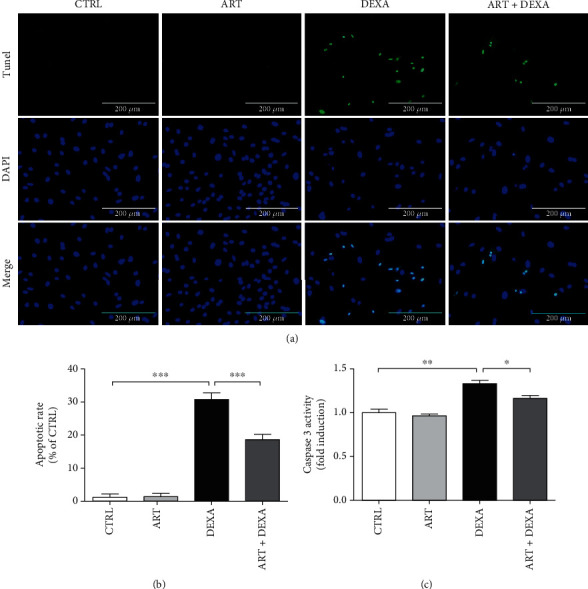
TUNEL/DAPI costaining and caspase-3 activity assays. Artemisinin attenuated DEXA-induced BMSC apoptosis. BMSCs were pretreated with 1.0 *μ*M artemisinin or DMSO (0.1%, vehicle control) for 1 h and then incubated with 30 *μ*M DEXA for 24 h. (a) Representative photomicrographs of BMSC DNA fragmentation and nuclear condensation after appropriate treatments as analysed by TUNEL/DAPI costaining assay (*n* = 3). (b) The apoptotic rate of BMSCs: quantitative analysis of (a) (*n* = 3). (c) Caspase-3 activity (*n* = 3). ^∗∗∗^*p* < 0.001, ^∗∗^*p* < 0.01, and ^∗^*p* < 0.05 were considered significantly different. CTRL: control; ART: artemisinin; DEXA: dexamethasone; ART + DEXA: treated with artemisinin followed by exposure to dexamethasone.

DNA fragmentation is an important event in the process of cellular apoptosis with caspases being crucial mediators of this cellular event. Among them, caspase-3 is a key protease that is frequently activated during the process of apoptosis. To further assess the protective effect of artemisinin against DEXA-induced apoptosis, TUNEL/DAPI costaining and caspase-3 activity assays were used. Obtained results revealed that DEXA-induced apoptosis of BMSCs was significantly attenuated by artemisinin pretreatment (Figure 4).

### 4.5. Artemisinin Attenuated DEXA-Induced *Δψ*m Loss

**Figure 5:**
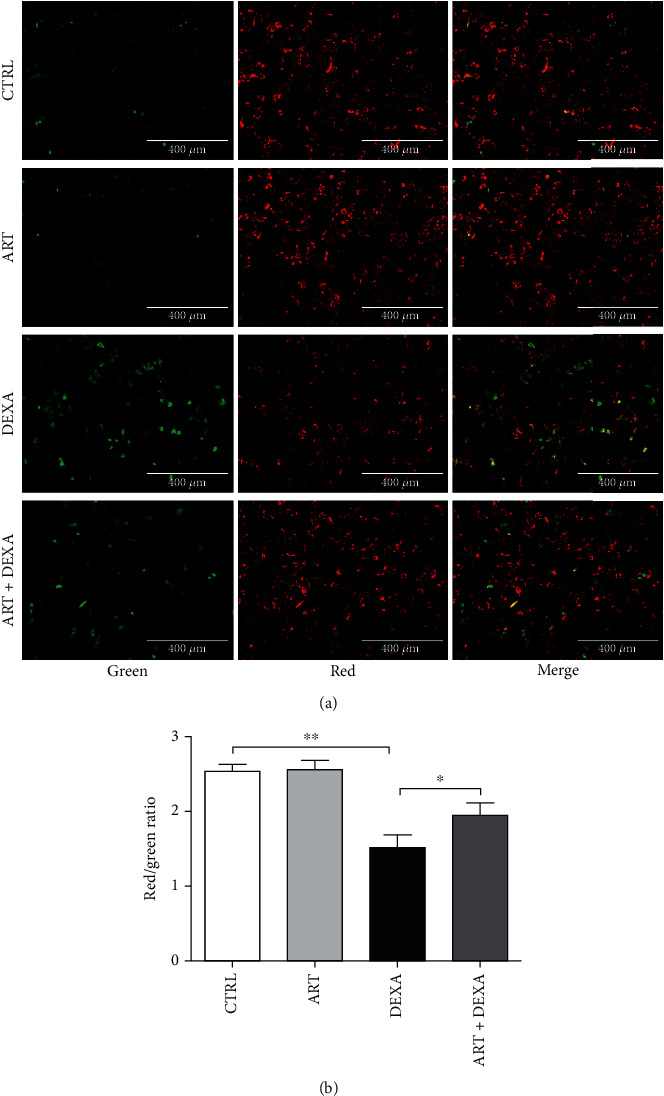
Artemisinin attenuated DEXA-induced *ΔΨ*m reduction. BMSCs were pretreated with 1.0 *μ*M artemisinin or DMSO (0.1%, vehicle control) for 1 h and then incubated with 30 *μ*M DEXA for 24 h. (a) *ΔΨ*m was analysed using JC-1 assay (*n* = 3). The reduction of *ΔΨ*m is reflected by the shift of fluorescence from red to green indicated by JC–1. (b) Red/green ratio quantification (*n* = 3). ^∗^*p* < 0.05 and ^∗∗^*p* < 0.01 were considered significantly different. CTRL: control; ART: artemisinin; DEXA: exposed to dexamethasone only; ART + DEXA: treated with artemisinin followed by exposure to dexamethasone.

Mitochondria are major intracellular organelles that manufacture energy molecules and play a critical role in the defense against oxidative stress-induced insults in many eukaryotic cells [32, 33]. The loss of *Δψ*m is also one of the most important events occurring in the early stages of apoptosis, being associated with the permeability of the mitochondrial membrane. For example, increased oxidative stress levels can damage the mitochondrial membrane and cause the transmembrane potential gradient to disappear. Therefore, *Δψ*m is the best indicator for monitoring mitochondrial inner membrane permeability and early cell apoptosis. JC-1 assay results revealed that DEXA significantly decreased the *ΔΨ*m of BMSCs in comparison with the control group. Artemisinin pretreatment prevented the loss of *ΔΨ*m induced by DEXA, which is indicative of its protective effect on the early stages of DEXA-induced apoptosis (Figure 5).

### 4.6. Artemisinin Reduced DEXA-Induced ROS Production

**Figure 6:**
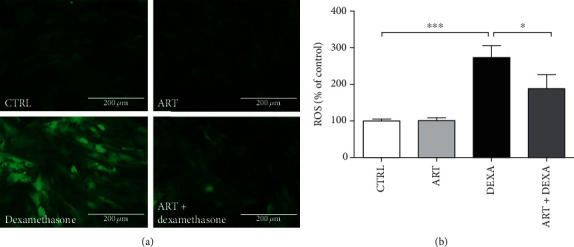
Artemisinin decreased intracellular ROS levels induced by DEXA. BMSCs were pretreated with 1.0 *μ*M artemisinin or 0.1% DMSO (vehicle control) for 1 h, followed by 24 h exposure to 30 *μ*M DEXA. (a) Fluorescent visualization of ROS production in BMSCs using CellROX™ Green fluorescence imaging. DEXA treated cells displayed deep green fluorescence, indicating increased ROS production (*n* = 3). (b) The quantitative analysis of the intracellular ROS levels. Bars show the average percentage of fluorescent positive cells out of the total number of cells (*n* = 3). ^∗^*p* < 0.05 and ^∗∗∗^*p* < 0.001 were considered significantly different. CTRL: control; ART: artemisinin; DEXA: dexamethasone; ART + DEXA: treated with artemisinin followed by exposure to dexamethasone.

The physiological levels of ROS can be used as key signaling molecules for cell proliferation and survival. However, excessive production of ROS surpasses the antioxidant ability of cells, rapidly triggering an oxidative stress response that causes direct or indirect damage to nucleic acids, proteins, and lipids [34]. Assessment of intracellular ROS levels using the CellROX™ showed a significant increase upon exposure to DEXA (273.35% of control) that was significantly attenuated by artemisinin treatment (188.61% of control) (Figure 6).

### 4.7. Artemisinin Improved Osteogenic Differentiation of DEXA-Damaged BMSCs

**Figure 7:**
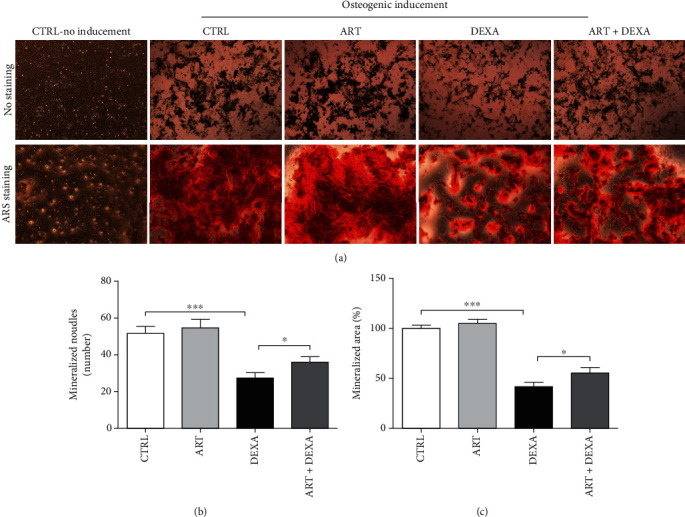
Artemisinin attenuated DEXA-inhibitory osteogenesis differentiation of BMSCs. P3 BMSCs were seeded in 6-well plates at the density of 4-6 × 10^4^ cells/mL, then treated with ART (1.0 *μ*M), DEXA (30 *μ*M), or ART (1.0 *μ*M) + DEXA (30 *μ*M) for 21 days. The formation of mineralized nodules was detected by ARS staining and observed using fluorescence microscopy (*n* = 3). (a) Representative images of ARS staining. (b) Quantification of the number of mineralized nodule number. (c) Quantification of the area in osteogenic differentiation using the ImageJ software (*n* = 3). ^∗^*p* < 0.05 and ^∗∗∗^*p* < 0.001 were considered significantly different. CTRL: control; ART: artemisinin; DEXA: dexamethasone; ART + DEXA: treated with artemisinin and then dexamethasone.

It has been recognized that low concentrations of DEXA positively regulate osteogenic differentiation, while high concentrations have an inhibitory effect [35, 36]. To further evaluate the protective effect of artemisinin against DEXA-induced alterations on BMSCs' osteogenic differentiation, cells were treated with ART, DEXA, or ART+DEXA for 21 days. ARS staining was used to detect the formation of mineralized nodules indicative of BMSCs' osteogenic differentiation. Obtained results revealed that the cells cultured in the presence of artemisinin alone for 21 days stained positive with no significant differences in comparison to the control group. In contrast, there was a significant decrease in the number of stained cells cultured in the presence of DEXA alone in comparison to the control group. Interestingly and importantly, the number of ARS positive cells cultured in ART+DEXA conditions was significantly higher than in the DEXA group. Notably, these data indicate that artemisinin treatment substantially rescued the osteogenic decline of BMSCs induced by a high dose of DEXA (Figure 7).

### 4.8. DEXA Inhibited the ERK1/2-CREB Signaling Pathway

**Figure 8:**
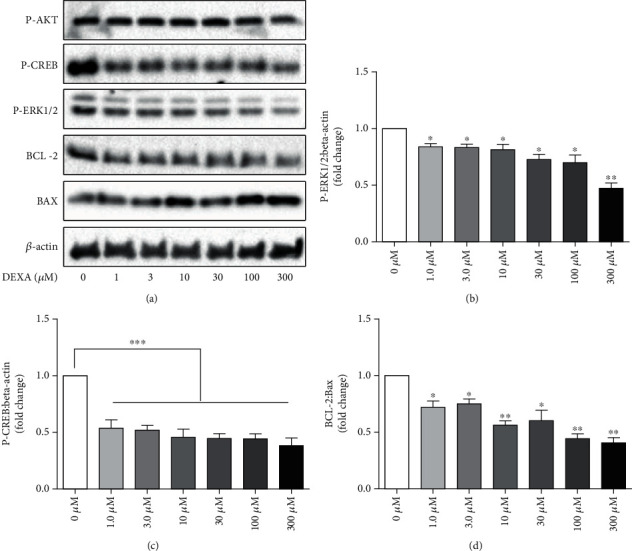
DEXA inhibited the ERK1/2-CREB signaling pathway. (a) BMSCs were seeded into a 6-well plate at a density of 1-2 × 10^5^ cells/mL and treated with different concentrations of DEXA (0-300 *μ*M) for 24 h. The expression levels of P-ERK1/2, P-CREB, P-AKT, BCL-2, and Bax were assessed by Western blot analysis (*n* = 3). (b–d) Quantification of (a). ^∗^*p* < 0.05, ^∗∗^*p* < 0.01, and ^∗∗∗^*p* < 0.001 were considered significantly different in comparison to the control group (0 *μ*M).

The ERK1/2-CREB pathway is a central signaling pathway that plays important roles in the regulation of many cellular processes, such as apoptosis, survival, proliferation, and differentiation. Assessment of the signaling pathways involved in the deleterious effect of DEXA on BMSCs revealed that the phosphorylation of ERK1/2 and CREB, but not AKT, was significantly decreased by DEXA (Figures 8(a)–8(c)). CREB is a major positive regulator of the antiapoptotic protein BCL-2 expression. Accordingly, the expression of BCL-2 and Bax ratio was significantly downregulated (Figures 8(a) and 8(d)). These data indicate that the damaging effect of DEXA on BMSCs may occur via inhibition of the ERK1/2-CREB signaling pathway.

### 4.9. Artemisinin Upregulated ERK1/2, CREB Phosphorylation, and BCL-2 Production in BMSCs

**Figure 9:**
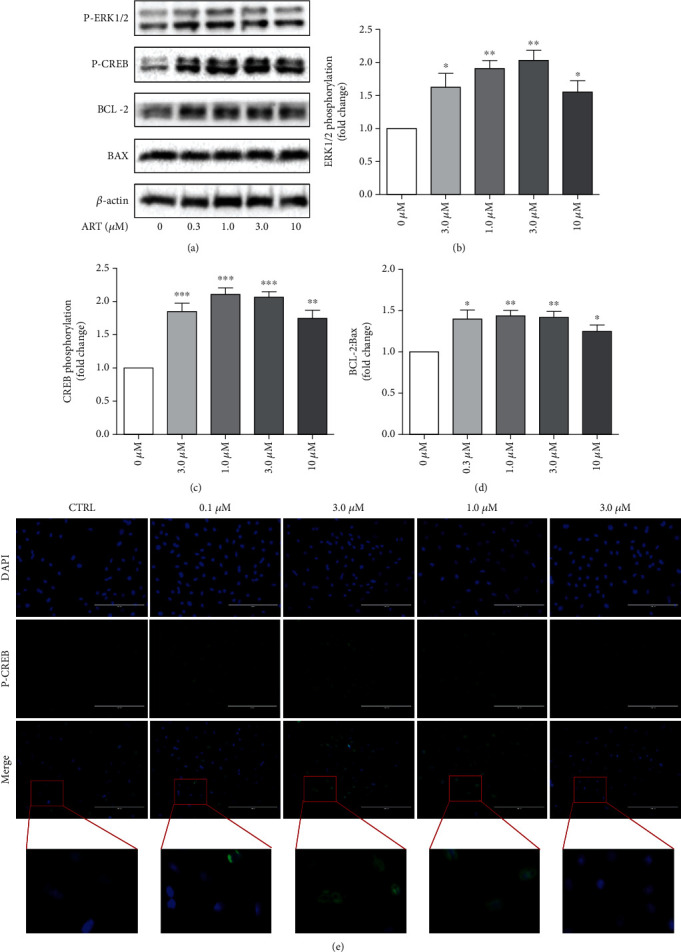
Artemisinin stimulated ERK1/2, CREB phosphorylation, and BCL-2 production. (a) BMSCs were seeded on 6-well plates at a density of 1-2 × 10^5^ cells/mL and treated with different concentrations of artemisinin for 1 h. The expression levels of P-ERK1/2, P-CREB, BCL-2, and Bax were assessed by Western blot analysis (*n* = 3). (b–d) Quantification of (a). (e) BMSCs were treated with different concentrations of artemisinin for 1 h, and the phosphorylation of CREB was detected my immunofluorescence (*n* = 3). ^∗^*p* < 0.05, ^∗∗^*p* < 0.01, and ^∗∗∗^*p* < 0.001 were considered significantly different in comparison to the control group (0 *μ*M).

We have previously reported that the ERK1/2 signaling pathway was involved in the protective action of artemisinin in rat BMSCs damaged by H_2_O_2_ [23]. As DEXA-induced BMSC damage promoted the inhibition of this pathway, we sought to investigate whether this pathway was also involved in the protective effect of artemisinin. To verify the hypothesis, BMSCs were treated with a variety of artemisinin doses, and the phosphorylation activities of ERK1/2, of the downstream target CREB, and of the antiapoptotic protein BCL-2 were measured. Obtained results revealed that the phosphorylation levels of ERK1/2 and CREB significantly increased after treatment with artemisinin (0.3 *μ*M to 30 *μ*M) for 1 h (Figures 9(a)–9(c)). Accordingly, the ratio of BCL-2/Bax was also significantly increased by artemisinin treatment (0.3 to 3 *μ*M) (Figures 9(a) and 9(d)). Further assessment of CREB activation by artemisinin by immunofluorescence (IFC) analysis confirmed that P-CREB was markedly upregulated by artemisinin, especially at 0.3 and 1.0 *μ*M doses (Figure 9(e)). These results demonstrate that artemisinin treatment promotes the activation ERK1/2-CREB pathway, suggesting its possible involvement in the protective action of artemisinin against DEXA-induced cellular damage in BMSCs.

### 4.10. ERK1/2 Was Involved in the Protective Effect of Artemisinin

**Figure 10:**
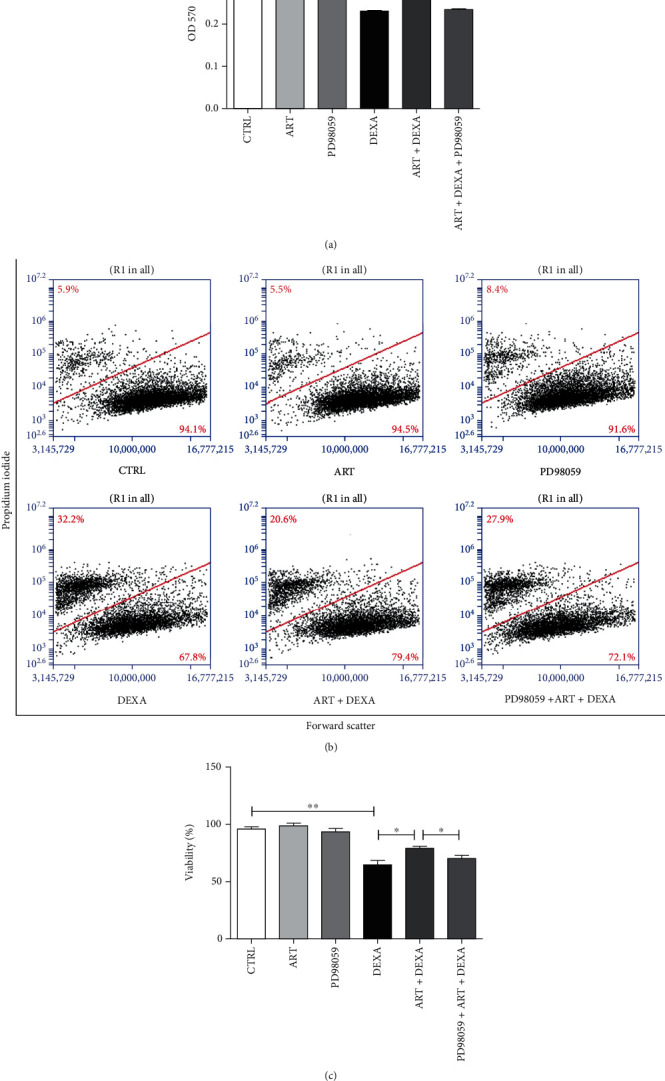
The protective effect of artemisinin was attenuated by ERK1/2 inhibitor PD98059. BMSCs were treated with 10 *μ*M PD98059 for 30 min. This step was followed by 1.0 *μ*M artemisinin treatment for 1 h and 30 *μ*M DEXA treatment for 24 h. The cell viability was detected by MTT assay (*n* = 4) (a) and by FACS using PI staining (*n* = 3) (b). (c) Quantification of (b). ^∗^*p* < 0.05 and ^∗∗^*p* < 0.01 were considered significantly different. CTRL: control; ART: exposed to artemisinin; DEXA: exposed to dexamethasone; ART + DEXA: treated with artemisinin followed by exposure to dexamethasone.

To confirm the involvement of ERK1/2 signaling in the protective effect of artemisinin against DEXA-induced damage, the inhibitor of ERK1/2 PD98059 was used to block ERK1/2 signaling. Cell viability analysis revealed that the protective effect of artemisinin was significantly inhibited by PD98059, strongly indicating the involvement of ERK1/2 (Figure 10(a)). These results were further confirmed by PI staining and FACS analysis to detect the cell viability (Figures 10(b) and 10(c)).

### 4.11. CREB Was Involved in the Protective Effect of Artemisinin

**Figure 11:**
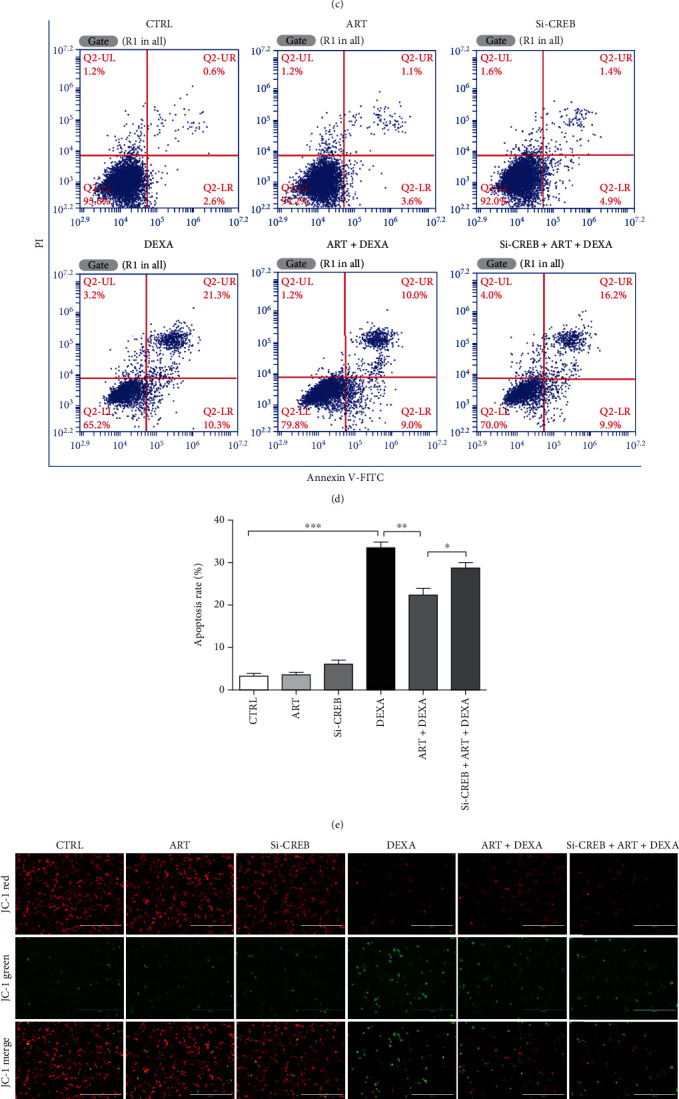
Protection of artemisinin was attenuated by siCREB. (a) BMSCs were transfected with 40 nM siCREB for 48 h, and the expression of T-CREB and GAPDH was detected by Western blot (*n* = 3). (b) Quantitative analysis of CREB relative expression using the ImageJ software (*n* = 3). ^∗∗∗^*p* < 0.001 versus control group was considered significantly different. BMSCs were transfected with 40 nM siCREB as above and pretreated with 1.0 *μ*M artemisinin for 1 h followed by incubation with 30 *μ*M DEXA for another 24 h. (c) Analysis of cell viability using MTT assay (*n* = 3). ^∗^*p* < 0.05 and ^∗∗∗^*p* < 0.001 were considered significantly different. (d) Analysis of apoptosis by FACS using Annexin V-FITC/PI staining (*n* = 3). (e) Quantitative analysis of (d). (f) Assessment of *Δψ*m using JC-1 assay (*n* = 3). (g) Quantitative analysis of (f) (*n* = 3). ^∗^*p* < 0.05, ^∗∗^*p* < 0.01, and ^∗∗∗^*p* < 0.001 were considered significantly different. CTRL: control; Si-NC: Si-negative control; ART: artemisinin; DEXA: dexamethasone; ART+DEXA: treated with artemisinin followed by exposure to dexamethasone: siCREB + ART + DEXA, cells were transfected with siCREB, then pretreated with artemisinin followed by incubation with dexamethasone.

The phosphorylation of ERK1/2 can activate its downstream signaling molecule CREB, which is an important intracellular protein that regulates the expression of proteins that are important in various physiological processes of BMSCs, including cell growth, proliferation, and differentiation [37–39]. To further investigate the role of the ERK1/2-CREB pathway in the protective action of artemisinin against DEXA-induced cellular damage, CREB was silenced by siCREB, and the cell viability, apoptosis, and *Δψ*m were assessed. CREB silencing significantly blocked the protective effect of artemisinin, resulting in a decrease of cell viability, increased apoptosis, and decreased *Δψ*m promoted by DEXA, strongly indicating the involvement of CREB in the protective effect of artemisinin against DEXA-induced cellular damage in BMSCs (Figure 11).

## 5. Discussion

**Figure 12:**
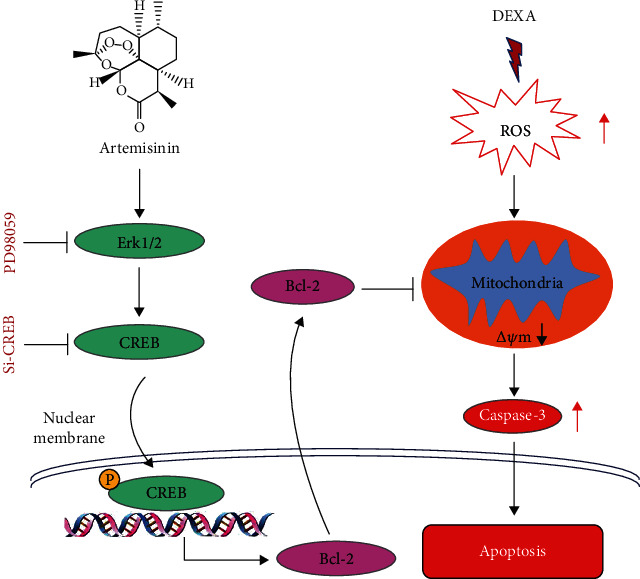
The blockage of ERK1/2 and CREB inhibited the protection provided by artemisinin in DEXA-damaged BMSCs. The blockage of ERK1/2 and CREB inhibited the protective effect of artemisinin against DEXA-induced damage, including apoptosis induction, ROS accumulation, and *Δψ*m loss. All the data strongly suggest that the protective effect of artemisinin against DEXA-induced damage in BMSCs is mediated, at least in part, by the ERK1/2-CREB signaling pathway.

This study demonstrates for the first time the protective effect of artemisinin on primary cultured rat BMSCs against glucocorticoid-induced cellular damage via activation of ERK1/2-CREB signaling pathway. In summary, we found that (1) high-dose DEXA (30 *μ*M) inhibited BMSC proliferation (Figures 1 and 2), promoted apoptosis (Figures 3 and 4), decreased *Δψ*m (Figure 5), and increased ROS production (Figure 6). (2) Assessment of the mechanisms involved on DEXA-induced apoptosis showed that DEXA downregulated the expression of P-ERK1/2, P-CREB, and BCL-2 (Figure 8). (3) Artemisinin protected BMSCs from high-dose DEXA-induced cellular viability decrease (Figure 2), apoptosis (Figures 3 and 4), caspase-3 activation (Figure 4), *ΔΨ*m loss (Figure 5), and intracellular ROS increase (Figure 6). (4) Blockade of ERK1/2 and CREB signaling by MEK/ERK inhibitor PD98059 or siCREB attenuated artemisinin protective effect (Figures 10–12). (5) High-dose DEXA inhibited BMSCs' osteogenic differentiation and treatment with artemisinin protected from DEXA-induced osteogenic differentiation damage (Figure 7).

DEXA is a synthetic glucocorticoid widely used in the clinic, reported to cause GIOP in cases of long-term use of high doses [13]. During the past years, the underlying mechanisms of DEXA-induced GIOP have been extensively studied with the most relevant comprising the ability of glucocorticoids to promote BMSC apoptosis, to stimulate adipogenic differentiation at the expense of osteoblastic differentiation, and to induce osteoblast and osteocyte apoptosis while promoting osteoclast differentiation and survival [40–42]. DEXA has been reported to have two diametrically opposite effects on osteogenic differentiation that are concentration dependent. While in physiological concentrations, glucocorticoids promote osteogenesis, high concentrations inhibit it and promote adipogenic differentiation increasing the risk of GIOP [22]. Therefore, glucocorticoid-inflicted damage on BMSCs is closely related to the initiation and progression of GIOP. Our research confirmed that DEXA has a dose-dependent effect on the viability of BMSCs, with high concentrations promoting the decrease of viable cells and the increase of cell death. In addition, we found that DEXA induced BMSC apoptosis, decreased *ΔΨ*m, and increased intracellular ROS. When treated with artemisinin, BMSC survival was significantly improved, and the effects of DEXA on intracellular ROS accumulation, caspase-3 activation, increased LDH release, *Δψ*m loss, and apoptosis were significantly prevented. In addition, artemisinin treatment substantially rescued the osteogenic differentiation decline of BMSCs induced by DEXA. In GIOP, the balance between osteogenic and adipogenic differentiation is broken as denoted by the decreased commitment of BMSCs to the osteogenic lineage at the expenses of adipogenic differentiation. As a result, the number of osteoblasts produced by BMSCs decreases, the number of adipocytes increases, and the adipocyte accumulation and osteoblast decrease cause OP due to the BMSCs inability to answer bone repair demands.

The ERK1/2-CREB pathway is widely involved in the processes of stem cell reproduction, survival, and differentiation. We found that DEXA hindered the proliferation of BMSCs, which is likely be related to its inhibitory effect on ERK1/2-CREB signaling pathway activation. In line with this, the blockade of ERK1/2 signaling has been previously reported to suppress osteogenic differentiation, favouring adipogenic differentiation and the expression of the peroxisome proliferator-activated receptor gamma 2 (PPAR*γ*2) and lipoprotein lipase [21, 37, 39]. Assessment of the mechanisms underlying the effect of artemisinin against DEXA indicates that its protective effect is likely to be related to the upregulation of this pathway. Inhibition of the ERK1/2-CREB signaling pathway with PD98059 and siCREB significantly attenuated the protective effect of artemisinin against DEXA insults, providing a potential mechanism involving the activation of this pathway on artemisinin action against DEXA-induced damage.

In summary, our findings point the use of artemisinin as a potential strategy for GIOP therapy. It allows the consolidation of the transient benefits of BMSC transplantation seen *in vivo* with artemisinin action improving the therapeutic outcomes of BMSC-based therapies for OP. Although GIOP cannot be completely defined as a stem cell disease, protecting these cells from DEXA-induced damage will have a great impact in the early prevention of GIOP. Hence, our findings provide a new strategy for GIOP therapy.

## Data Availability

The data used to support the findings of this study are included within the article.
